# Consequences of hyposalivation in relation to cancer treatment and early management of radiation-induced caries: case reports

**DOI:** 10.1038/s41415-024-7894-6

**Published:** 2024-11-08

**Authors:** Delphine Maret, Natalia Ranger Palmier, Philippe Guignes, Sabine Betancourt, Marie-Christine Teulières, Emmanuelle Vigarios, Avijit Banerjee

**Affiliations:** 41415252865001 Département d´Odontologie, Université Paul Sabatier, Service d´Odontologie, Centre Hospitalier Universitaire, Laboratoire Centre d´Anthropobiologie et de Génomique de Toulouse, Toulouse, France; 41415252865002 Dental Oncology Service, Instituto do Câncer do Estado de São Paulo (ICESP-FMUSP), São Paulo, Brazil; 41415252865003 Département d´Odontologie, Université Paul Sabatier, Service d´Odontologie, Centre Hospitalier Universitaire, Toulouse, France; 41415252865004 Département de Médecine Orale, Institut Claudius Regaud, Institut Universitaire du Cancer Toulouse-Oncopole, France; 41415252865005 Département de Médecine Orale, Oncopole Claudius Regaud, Institut Universitaire du Cancer Toulouse-Oncopole, Toulouse, France; 41415252865006 Professor of Cariology & Operative Dentistry and Honorary Consultant, Restorative Dentistry, Faculty of Dentistry, Oral & Craniofacial Sciences, King´s College London, UK; Honorary Consultant Advisor, Office of the Chief Dental Officer, England, UK

## Abstract

Given the prevalence of head and neck carcinoma and the salivary changes induced by the oral side effects of radiotherapy, such patients are at higher risk/susceptibility of developing dental caries. Radiation-caries is often under-treated in patients undergoing cervicofacial radiotherapy, yet these lesions can increase the risk of osteoradionecrosis due to necessary subsequent dental extractions. Moreover, xerostomia is accompanied by difficulties with speech, chewing and swallowing. The prevention and/or early management of radiation-induced carious lesions is essential in preventing oral complications and improving patients' quality of life. Based on a French case series, this paper proposes a protocol for the dental management of radiation-induced carious lesions.

## Key points

- Demonstrates that patients receiving cervicofacial radiotherapy who develop hyposalivation should undergo active surveillance at an early stage in order to prevent/limit the progression of caries.
- Suggests that professional application of fluoride varnishes is an alternative to be considered if trays cannot be worn.
- Applying silver diamine fluoride during cancer treatment not only prevents the formation of new carious lesions, but also halts the progression of existing ones, particularly on proximal root surfaces where access is limited for diagnosis and restoration.

## Introduction

The increasing survival rate of head and neck cancer patients makes the prevention and management of oral sequelae increasingly relevant in primary care. One of the most frequent is radiation-induced dental caries, which can lead to tooth extractions and, potentially, osteoradionecrosis of the jaws.^1^^,^^2^ Although it is thought that ionising radiation causes direct damage to the dentition, particularly when cervicofacial radiotherapy doses exceed 60 Gy,^3^^,^^4^^,^^5^ this belief has been challenged on the basis that most of the studies have been performed *in vitro.*^6^ The oral changes that contribute to the development of radiation-induced carious lesions have instead been attributed to indirect effects due to a cluster of oral symptoms,^7^ such as oral mucositis, dysgeusia, trismus, hyposalivation due to damage to the major salivary glands, and a reduction in salivary buffering capacity to a pH below that at which enamel begins to demineralise.^8^^,^^9^

Chronic hyposalivation can increase the risk of oral infections and carious lesions. The xerostomia experienced by patients is accompanied by difficulties with speech, chewing and swallowing. Patients presenting with hyposalivation as a result of cervicofacial radiotherapy are at higher risk/susceptibility of dental caries.^1^ Dry mouth promotes the growth of cariogenic flora, the reduction of antimicrobial salivary protein concentrations and the loss of mineralising salivary components. These conditions are conducive to the development of carious lesions, which may be preceded by dentine sensitivity.^10^^,^^11^ Radiation-induced alterations of salivary composition make teeth more susceptible to demineralisation, hence affecting their mineral structure.^10^^,^^12^^,^^13^^,^^14^

Radiation-induced caries is a complication of cervicofacial radiotherapy that affects over 30% of patients in the first year of treatment.^15^^,^^16^^,^^17^^,^^18^^,^^19^ Radiation-induced carious lesions have a unique progression profile and particularly affect the cervical areas, particularly around the mandibular anterior teeth and incisal free edges, cusps and smooth surfaces, which are generally unaffected by ‘conventional' carious lesions.^11^ The disease course starts by enamel decalcification (chalky white and brown decalcification).^1^^,^^20^ Radiation-induced carious lesions differ from conventional ones in their ability to spread rapidly. Furthermore, they are very often asymptomatic until their late-stage presentation.^15^^,^^16^^,^^17^^,^^18^^,^^19^

Treatment is a challenge for primary care clinical practitioners. The absence of established protocols can lead to high rates of restorative failure, persistent foci of infection and a higher risk of post-extraction osteoradionecrosis and hospitalisation. There are no clearly published recommendations or validated protocols, so early patient management remains challenging. The term ‘post-radiation caries' derives from the fact that carious lesions are usually clinically visible within a year after the end of radiotherapy. However, the caries process starts as soon as the salivary changes induced by cancer treatment occur and the salivary changes also exacerbate existing carious lesions.

Patients should be offered management strategies following the team-delivered, person-focused, susceptibility-related, prevention-based, minimum intervention oral care (MIOC) delivery framework as soon as possible,^21^^,^^22^^,^^23^^,^^24^^,^^25^^,^^26^^,^^27^^,^^28^^,^^29^ without waiting for anticancer therapy to be completed.^30^^,^^31^^,^^32^ In addition, the anatomical location of these lesions often makes minimally invasive operative intervention difficult, again emphasising the importance of early preventive non-operative therapies.

Different remineralisation technologies are available. Remineralising agents, such as fluoride, have a place in the general management of caries in highly susceptible patients and can be used both for the prevention and treatment of incipient lesions.^32^ Topical fluorides are rich in fluoride, typically containing 22,600 ppm. When a fluoride varnish is applied, a dense, labile layer of calcium fluoride is formed. As part of the remineralisation process, this layer serves as a reserve of fluoride and calcium, which are released when the pH becomes acidic. This controlled release contributes to the remineralisation process.^33^^,^^34^ With the silver diamine fluoride (SDF) solutions, silver ions interact with dentine proteins to form bacterio-resistant complexes (increased hardness and slower biofilm formation).^35^^,^^36^^,^^37^ The presence of silver turns the lesion black by reducing silver ions to metallic silver and silver oxides. SDF varnish is an effective medical product and its use is relevant even on advanced lesions, either alone or in combination with a restorative procedure.^35^^,^^36^^,^^37^

This paper reports two clinical cases that highlight two consequences of radiation-induced hyposalivation on dental tissues: enamel decalcification ie chalky white and brown decalcification, associated with reported dentine hypersensitivity; and clinically detectable radiation-induced carious lesions.

## Case 1

A 48-year-old woman was referred to the oral medicine department of IUCT-Oncopole (Toulouse, France) by their medical team owing to acute diffuse pain in all teeth that had begun one month earlier. She was first seen at the IUCT-Oncopole as part of a follow-up of a squamous cell carcinoma of the mobile lateral border of the tongue, detected in May 2023 by a private maxillofacial surgeon. She was treated with primary surgery (marginal glossectomy, cervical lymph node curettage) followed by linear accelerator-based intensity-modulated radiation therapy (IMRT) for seven weeks (70 grays [ie 2 Gy/day]) administered in a cancer centre in the Toulouse region.

The MIOC delivery framework is divided into four interlinked clinical domains: identification of the problem (detection, susceptibility assessment, special investigations, diagnosis, phased personalised care planning with shared-decision making); prevention of lesions/control of disease (patient behaviour change, non-operative, micro-invasive interventions); minimally invasive operative interventions; and recall/re-assessment/active surveillance. This was used to structure the management of the patient.^38^

### MIOC Domain 1 - identifying the problem

#### Medical and dental history

- Former smoker (ten packs/year), weaned off 2022
- No current medication
- No known allergies
- Reason for consultation: acute diffuse dental sensitivity one month post-radiotherapy. The patient expressed concern about the future of her teeth. Her dental follow-up had been irregular throughout her life and non-existent for around two years previously.

#### Clinical examination

**Fig. 1:**
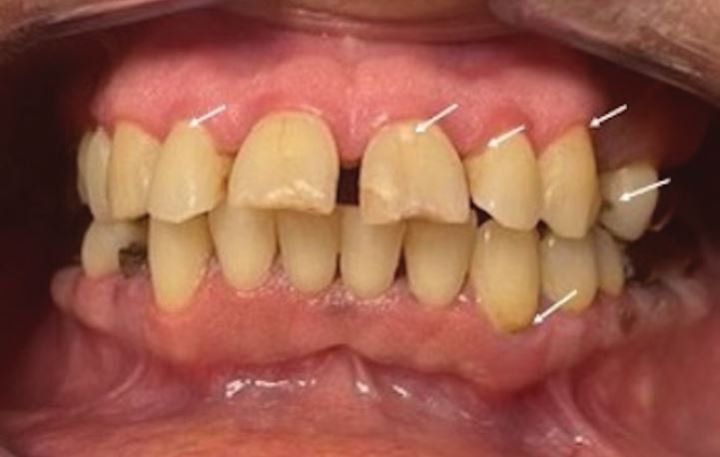
An anterior clinical view of the patient in intercuspal position, showing the oral and dental status one month post-cervicofacial radiotherapy with dental demineralisation with white and brown stains (arrows)

Clinical examination revealed pain associated with thermal, air and tactile stimuli. According to the Common Terminology Criteria for Adverse Events v.5, the severity of xerostomia was Grade 2. She presented the following: dental demineralisation with white and brown stains at the cervical level of teeth 12, 21, 22 and 33, and at the free edge of the tooth 21; arrested carious lesions on the proximal surfaces of teeth 11, 12, 21 and 24; gingivitis induced by plaque biofilm and associated with dry mouth (local risk factor); root remnants of teeth 16 and 44 without associated symptoms (palpation, percussion) or signs of infection; teeth 24, 27, 28 and 47 missing; and difficulty keeping her mouth open. No radiograph was available. See Figure 1.

#### Diagnosis

- Enamel decalcification (chalky white and brown decalcification), first stage of radiation-induced caries^1^
- The objective was to prevent the onset of radiation-induced lesions, which initially manifest as enamel demineralisation with the appearance of white or brown spots on the cervical, incisal edges or free margins. The symptomatology described by the patient was suggestive of this process.^1^

### MIOC Domain 2 - prevention and control

The phased, personalised care plan proposed to and agreed with the patient was as follows:

- Suitable effective oral hygiene
- Daily application of topical fluoride (ie twice-daily brushing with a minimum of 5,000 ppm fluoride toothpaste [Duraphat; Colgate, or Fluodonty Cooper])
- Wearing fluoride trays containing 20,000 ppm fluoride gel (Fluogel MOC) for five minutes a day
- Increase the frequency of oral hygiene reminders/assessments and active surveillance of patient-described symptomatology (15 days after the first appointment, then one month, then every three months; once stability achieved, every six months).

Oral hygiene assessment, plaque presence and dietary survey were systematically investigated.

### MIOC Domain 4 - re-assessment/active surveillance

**Fig. 2:**
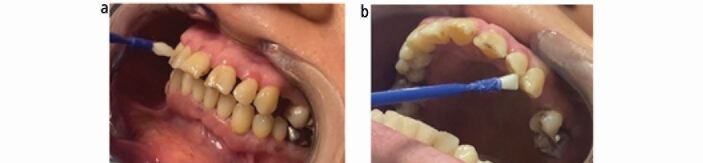
a, b) Topical application of fluoride varnish to both arches, all dental surfaces after careful drying of the surfaces

**Fig. 3:**
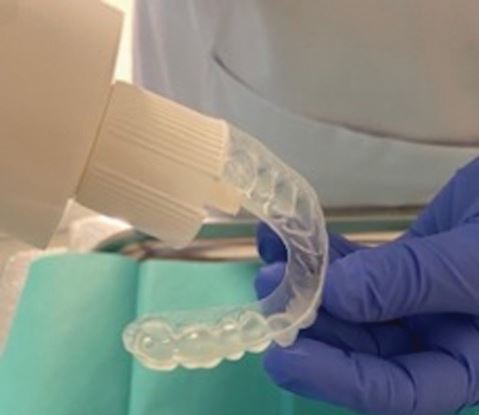
Example of a fluoride tray

The first recall visit took place two weeks after the initial appointment, with application of fluoride varnish (Fig. 2) and impressions taken for the fluoride trays. The patient noted a marked improvement with a reduction in dentine sensitivity. Oral hygiene assessment, plaque presence and dietary survey were systematically re-investigated. One month later, fluoride trays and associated recommendations were given. Dental sensitivity symptoms were also investigated. (Fig. 3). The patient was now pain-free. A review consultation was planned at three months and then, in the future, in primary care.

## Case 2

**Fig. 4:**
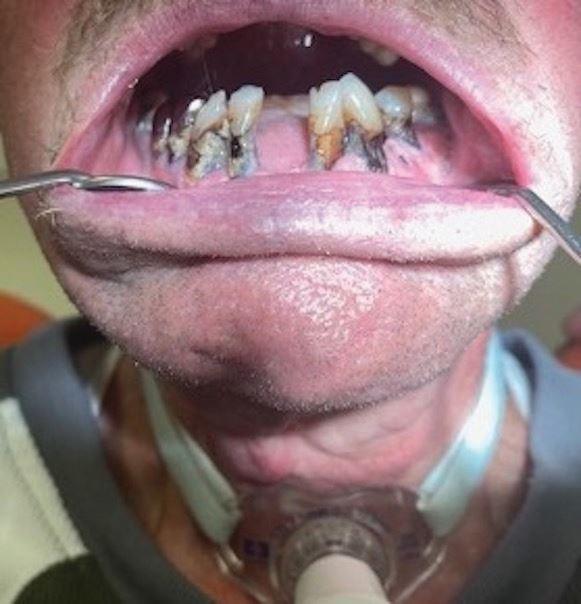
Initial oral status showing a total absence of dental clinical follow-up. The clinical examination shows the presence of plaque related to lack of oral hygiene, periodontitis without tooth mobility and the presence of dental caries

A 69-year-old man was referred by his medical team because of his poor oral and dental condition, associated with xerostomia following anticancer treatment and a total absence of dental clinical follow-up for more than five years (Fig. 4). He was first seen at the IUCT-Oncopole (Toulouse, France) for follow-up of retro-cricoarytenoid squamous cell carcinoma in September 2023. Treatment was as follows: neoadjuvant chemotherapy with tracheostomy in 2023 followed by linear accelerator-based IMRT for seven weeks (70 grays [ie 2 Gy/day]).

### MIOC Domain 1 - identifying the problem

#### Medical and dental history

- Squamous cell carcinoma of the free edge of the right tongue. Operated in 2020 and associated with cervicofacial radiotherapy in a cancer centre in the Toulouse region
- Alcohol withdrawal six months before
- Former smoker (20 packs/year), weaned off 2021
- No current medication
- No known allergies
- Referred by the medical team for his oral condition, xerostomia and lack of dental follow-up for over five years. The patient reported no dental pain but wondered about the discoloration and rapid deterioration of his teeth in recent years. He confided that he did ‘not want to invest time and money' in his mouth.

#### Clinical examination

**Fig. 5:**
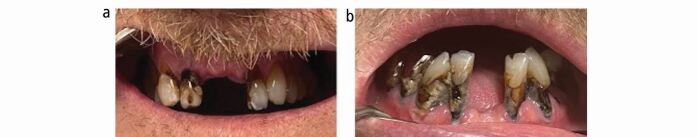
a, b) Presence of cervical and root carious lesions on teeth 13, 12, 22, 32, 33, 35, 37, 42, 43, 44, 45 and 47. Presence of carious lesions on free edge of 32

He presented the following: cervical and root carious lesions on teeth 13, 12, 22, 32, 33, 35, 37, 42, 43, 44, 45 and 47; a carious lesion on the free edge of the 32; plaque related to lack of oral hygiene; periodontitis without tooth mobility; missing teeth 11, 17, 18, 21, 27, 28, 31, 37, 38 41, 46 and 48; amalgam restorations of the 15, 25, 35, 36 and 47; single-unit metal crown prosthesis of the 44 and 45; and difficulty keeping his mouth open. See Figure 5.

#### Radiographic examination

See Figure 6.

**Fig. 6:**
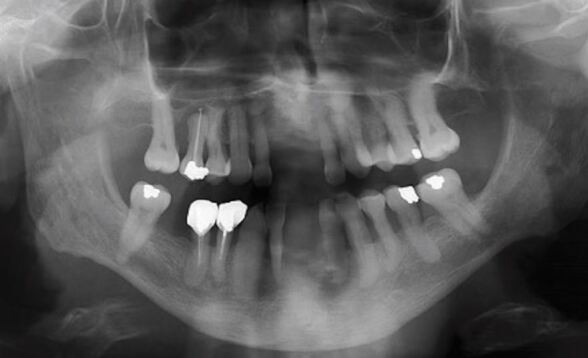
Dental panoramic showing the following radiographic details: endodontic treatment on 15, 44, and 45; presence of periapical radiolucency of the 15 associated with endodontic treatment without associated symptomatology; and bone loss associated with root surface exposure

- Endodontic treatment: 15, 44 and 45
- Presence of peri-apical radiolucency of 15 associated with endodontic treatment without associated symptomatology
- Bone loss associated with root surface exposure.

#### Diagnosis

- In relation to dental hard tissue: radiation-induced carious lesions
- Management was focused on halting the progression of the radiation-induced carious lesions and preventing the development of new lesions. Progression of lesions can lead to coronal fracture and increases the risk of osteoradionecrosis post-extraction. Given the palliative context of this personalised care plan and in agreement with the patient, no prosthetic rehabilitation was proposed.

### MIOC Domain 2 - prevention and control

The phased personalised care plan proposed to and agreed with the patient was as follows:

- Recommendations for improved oral hygiene
- Application of SDF to maintain the remaining teeth
- Recall/active surveillance frequency every month for three months.

The review consultations were particularly important in this case as the objective was to maintain the patient's existing dentition without resorting to operative interventions (MIOC Domain 3). The persistent, non-symptomatic, chronic pathology associated with both radiation-induced carious lesions and periodontal disease observed in this case made this patient highly susceptible to osteoradionecrosis if further invasive surgery was carried out.

### MIOC Domain 4 - review/active surveillance

**Fig. 7:**
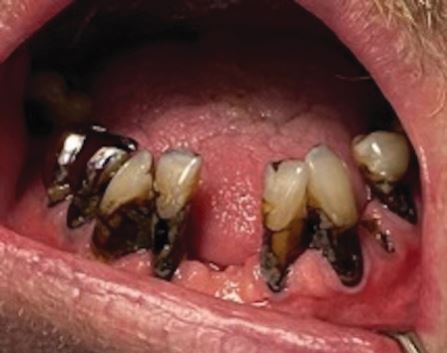
Inspection six months after first application of SDF. Improved oral hygiene and stabilisation of carious lesions

The patient was recalled every month during the first six months of follow-up (September 2023 to March 2024) to review his oral hygiene status and stabilisation of the caries process, and ensure there was no sign of infection or symptomatic teeth. At six months, his oral condition was stable with no associated symptoms. His oral hygiene had improved considerably (Fig. 7), despite his state of physical and psychological fatigue.

## Discussion

Reduced salivary flow as a consequence of cervicofacial radiotherapy leads to changes in the oral bacterial flora and may lead to an increase in cariogenic bacteria in saliva.^11^ After identifying, diagnosing and appropriate care planning (MIOC Domain 1), the first step is to care for the patient's oral hygiene, which is essential for minimising the impact of cariogenic microflora. According to Pinna *et al.*^12^ and in line with best practice, head and neck cancer patients treated with radiotherapy and presenting with hyposalivation could constitute a new risk group for dentine sensitivity or hypersensitivity caused by enamel demineralisation. Management of these patients should focus on preventive measures, such as maintaining oral hygiene during radiotherapy.^39^^,^^40^^,^^41^ Among the non-invasive therapies, the application of desensitising varnishes leads to a reduction in symptoms in irradiated patients. The varnishes contain fluoride, which has a preventive topical effect during radiotherapy. Once radiotherapy is over, a fluoride gel is applied daily at home via custom-made thermoformed dental trays to prevent the development of carious lesions.^18^^,^^19^^,^^20^^,^^30^ If patients are unable to wear the trays owing to disability or lack of behavioural adherence, regular professional applications every three months may be suggested.

Finally, the patient is informed, facilitated and encouraged as to how to deal with xerostomia and to ensure optimal oral hygiene, prevent caries and manage dentine sensitivity. To limit dentine hypersensitivitý, it is recommended to use an arginine- or tin-fluoride-based toothpaste with brushing twice daily, followed by digital application to any painful areas for one minute.

If the patient has radiation-induced carious lesions, management should be aimed at limiting the progression of the disease and preventing the risk of osteoradionecrosis by applying SDF varnish. This ‘palliative' management seeks to improve the patient's quality of life. Treatment is therefore limited to topical applications of SDF to halt caries progression, while ensuring that there are no associated symptoms or signs of infection. The application of SDF during cancer therapy not only prevents the formation of new carious lesions but also halts the progression of existing ones, particularly on root proximal surfaces where access is limited for diagnosis and restoration. Clinical studies on patients treated with cervicofacial radiotherapy to prevent and limit the development of radiation-induced carious lesions are still lacking, as are consensual recommendations. It is also important to remember that the minimally invasive procedure, although helpful, does not substitute active caries tissue removal and restoration. Each case should be addressed individually in terms of pros and cons and patients' ability to comply both financially and with ‘at home personal care'.

## Conclusion

These cases demonstrate that patients receiving cervicofacial radiotherapy who develop hyposalivation should undergo active surveillance at an early stage in order to prevent/limit the progression of caries. Using the MIOC delivery framework, they should be given advice/knowledge on the following: a) avoiding/limiting the dentine sensitivity resulting from enamel demineralisation and dentine exposure; b) preventing caries, in particular during the early phase of radiation-induced carious lesions; c) understanding the course of radiation-induced carious lesions; d) implementing non-invasive therapeutic solutions during and after cancer treatment; and e) coping psychologically with xerostomia and undertaking appropriate oral hygiene.
